# Neurobehavioral Effects in HIV-Positive Individuals Receiving Highly Active Antiretroviral Therapy (HAART) in Gaborone, Botswana

**DOI:** 10.1371/journal.pone.0017233

**Published:** 2011-02-18

**Authors:** Kathy Lawler, Kealeboga Jeremiah, Mosepele Mosepele, Sarah J. Ratcliffe, Catherine Cherry, Esther Seloilwe, Andrew P. Steenhoff

**Affiliations:** 1 Center for the Study of HIV and AIDS, University of Botswana, Gaborone, Botswana; 2 Infectious Disease Care Center, Princess Marina Hospital, Gaborone, Botswana; 3 Center for AIDS Research and Botswana-UPenn Partnership, University of Pennsylvania, Philadelphia, Pennsylvania, United States of America; 4 Department of Neurology, University of Pennsylvania, Philadelphia, Pennsylvania, United States of America; 5 Department of Biostatistics and Epidemiology, University of Pennsylvania, Philadelphia, Pennsylvania, United States of America; 6 Department of Medicine, University of Pennsylvania, Philadelphia, Pennsylvania, United States of America; 7 Children's Hospital of Philadelphia, Philadelphia, Pennsylvania, United States of America; 8 Monash University, Melbourne, Victoria, Australia; 9 Burnet Institute, Centre for Virology and Alfred Hospital, Department of Infectious Diseases, Melbourne, Victoria, Australia; University of Cape Town, South Africa

## Abstract

**Objective:**

To explore the prevalence and features of HIV-associated neurocognitive disorders (HANDS) in Botswana, a sub-Saharan country at the center of the HIV epidemic.

**Design and Methods:**

A cross sectional study of 60 HIV-positive individuals, all receiving highly active antiretroviral therapy (HAART), and 80 demographically matched HIV-seronegative control subjects. We administered a comprehensive neuropsychological test battery and structured psychiatric interview. The lowest 10^th^ percentile of results achieved by control subjects was used to define the lower limit of normal performance on cognitive measures. Subjects who scored abnormal on three or more measures were classified as cognitively impaired. To determine the clinical significance of any cognitive impairment, we assessed medication adherence, employment, and independence in activities of daily living (ADL).

**Results:**

HIV+ subjects were impaired for all cognitive-motor ability areas compared with matched, uninfected control subjects. Thirty seven percent of HIV+ patients met criteria for cognitive impairment.

**Conclusion:**

These findings indicate that neurocognitive impairment is likely to be an important feature of HIV infection in resource-limited countries; underscoring the need to develop effective treatments for subjects with, or at risk of developing, cognitive impairment.

## Introduction

Botswana has one of the highest human immunodeficiency virus (HIV) prevalence rates in the world, but it also has one of the most comprehensive nationally organized HIV testing and antiretroviral (ARV) therapy programs within sub-Saharan Africa [1]. Despite the initial success in Botswana in providing comprehensive free HIV testing and treatment with ARVs, there have been no studies to assess possible neurobehavioral complications in HIV-positive (HIV+) individuals on highly active antiretroviral treatment (HAART). In this paper we present the first systematic survey describing neurocognitive and affective functioning related to HIV infection in a HAART-treated Batswana cohort, where cade C is the predominant subtype.

It has been well documented that HIV enters the central nervous system (CNS) early after infection [2], [3] and that as many as 50% of individuals with AIDS have neurocognitive impairment [4]. Even mild neuropsychological impairment has been associated with decreased vocational functioning, problems with medication management, driving, and activities of daily living [5], [6]. Almost all of these data come from studies conducted in the United States [7], [8] Europe [9], and the Pacific Region/Australia [10]. Less is known about the prevalence and characteristics of neurobehavioral complications of HIV infection in developing African countries such as Botswana, which carry the burden of the HIV epidemic [11]–[14]. Research has suggested that the incidence of HIV-associated neurocognitive disorder (HAND) may vary due to different viral clades. For example, in Uganda clades A and D are the predominant subtypes, and a study found 31% of HIV+ individuals had dementia, and 47% mild cognitive impairment [15]. An additional study in Uganda showed that risk factors for HAND were low CD4 cell count and advanced age [13]. In contrast, a study in Ethiopia, which is primarily clade C, did not find cognitive impairment on a screening measure of dementia in HIV+ subjects compared with control subjects [12]. Another study of clade C HIV+ subjects in several Pacific Rim countries found cognitive impairment (verbal fluency 33.6%), decreased fine motor speed (finger tapping 43%), and impaired gait (28%) [10]. Similarly, a study in southern India, with predominantly clade C HIV+ subjects, found mild to moderate cognitive impairment in 60.5% of subjects with no clinically identified functional impairment [16]. A study in China indentified cognitive impairment in 34.2% of HIV+ subjects, with cognitive impairment in 39.7% of subjects who were co-infected with HIV and hepatitis C virus [17]. Most recently a study in South Africa, which is also predominantly clade C, found a prevalence of 42.4% mild cognitive disorder and 25.4% dementia in HIV+ patients beginning anti-retroviral therapy. Risk factors included lower levels of education, older age, and male gender [18].

While HAART has had a dramatic effect on the incidence and severity of HAND, its milder form continues to be prevalent. This may be due to a number of factors that include: delayed initiation of HAART, poor CNS penetration of many ARVs, drug resistance, potential ARV neurotoxicity, poor medication adherence, side-effects of long-term use of HAART, such as cardio-vascular disease, and chronic HIV brain infection [19], [20].

Diagnosis of HAND is of great importance, not only clinically, but also to ensure appropriate allocation of scarce medical resources in the regions worst affected by the HIV epidemic. To address this, the aims of the present study were twofold: 1) To determine the prevalence of cognitive impairment in an HIV-positive population in Botswana, a country with one of the highest HIV rates in the world, and 2) To assess, from a neurocognitive perspective, the effectiveness of HAART regimens with differing CNS penetration scores for HIV-positive patients in a resource-limited setting.

## Methods

### Ethics Statement

This research study was approved by the Institutional Review Boards (IRBs) from the Botswana Ministry of Health, Princess Marina Hospital (PMH), University of Pennsylvania, and Monash University. Written informed consent was obtained from the participants after the study had been fully explained to them.

HIV+ Participants: Sixty HIV+ subjects were asked to participate. They were approached from March through August 2009 during routine follow-up visits at the Infectious Disease Care Clinic (IDCC) at PMH in Gaborone, Botswana. A small reimbursement ($4.00) for participation was offered. Inclusion criteria were men and women with: (1) documented HIV-positive status; (2) ages 21–50; (3) ambulatory status; and (4) the ability to comprehend study procedures and provide informed consent. All patients were prescribed HAART. Exclusion criteria eliminated individuals with cognitive impairment unrelated to HIV, such as: (1) neurological conditions (e.g. head injury, seizure disorder); (2) chronic medical illness unrelated to HIV (e.g. chronic hepatic or renal failure, malignancy) or severe HIV-related disease (current opportunistic infection); (3) current fever; (4) severe psychiatric disorder (e.g. schizophrenia); (5) a history of substance abuse; (6) sensory impairment (e.g. deafness) that would impede understanding of test directions; or (6) inability to function independently as assessed using the study instruments.

### Control Subjects

Eighty demographically matched HIV-negative (HIV-) individuals who met the inclusion/exclusion criteria, also agreed to participate. All controls received a negative ELISA HIV test on the day of neurobehavioral testing, and were recruited from Tebelopele Voluntary Counseling and Testing Centre (TVCTC), a free government sponsored HIV testing centre adjacent to PMH.

### Procedure

Subjects underwent a standardized neuropsychological examination, a structured psychiatric interview of depression/anxiety, and an assessment of activities of daily living (ADL). Assessments were in the preferred language (Setswana or English) for each subject, administered by a Motswana medical student fluent in both languages (K.J.) or a neuropsychologist from the University of Pennsylvania (K.L.). Testing procedures were standardized for these two examiners. The neuropsychologist thoroughly trained and closely supervised the medical student. All consent forms, questionnaires, and tests were translated into Setswana and back translated. For patients with limited reading ability, the ADL scale was administered orally by the examiners. The investigators evaluated neuropathy using a targeted history composed of structured validated questions designed to elicit symptoms of distal sensory loss or pain. Structured interviews and chart reviews were performed to obtain information about medical and psychiatric history, pattern of substance use, adherence to ARV medication, marital status, education, employment, current medications, current and nadir CD4 lymphocyte counts, and viral loads. The assay used for viral load testing in Botswana is the Amplicor HIV-1 Monitor Assay, Roche Molecular Systems, Branchburg, New Jersey. The lower limit of detection of the assay is 400 copies/mL. Viral loads were categorized dichotomously as detectable (>400 copies/ml) versus undetectable (<400 copies/ml).

### Measures

The neuropsychological test battery assessed six cognitive-motor ability areas: processing speed, psychomotor speed, verbal learning/memory, executive function, language, and fine motor speed. Research has shown that these ability areas are consistently affected by HAND in developed countries [21]–[23] and more recently in several resource-limited countries [14], [16], [17], [24]–[26]. This battery was primarily made up of tests that have been widely used to study HIV infection in the United States, Europe, and multi-national studies in developing regions [19], [23]. In addition, we included a modified auditory verbal learning test, The Botswana Auditory Verbal Learning Test (BAVLT). This test was designed for administration in either Setswana or English, and is more appropriate for use in Botswana than existing verbal learning tests [27].

Participants underwent structured psychiatric interviews to assess major depressive disorder (MDD), generalized anxiety disorder (GAD), and alcohol use. To explore the clinical significance of neuropsychological impairment, we assessed independence in activities of daily living (ADL), employment status, and adherence to ARV regimens.

### Digit Symbol Coding (DSC) sub-test of the WAIS-III [28]

The DSC is a paper-pencil measure of processing speed. Subjects use a key of digit-symbol pairs at the top of the test page and are required to fill in the correct symbol for each number as quickly as possible within a 120 second time limit. The score is the number of correct symbols transcribed within the time limit.

### Verbal Fluency for Action

Action fluency (AF) is a test of language that taps into frontal neural systems [29] and has been shown to be sensitive to the neurocognitive effects of HIV infection in subjects in both developed and resource-limited countries [30]. This task requires subjects to generate as many action words (verbs) as they can within a time limit. The score is the total number of correct words produced within one minute.

### Botswana Auditory Verbal Learning Test (BAVLT)

The BAVLT [27] is similar to other verbal learning tests, including the WHO/UCLA Auditory Verbal Learning Test, which has been shown to be sensitive to HIV in several developing countries [16], [22], [31]. The BAVLT is a list-learning task, composed of 16 words from 4 categories, and an interference word list (16 different words, 4 different categories). This test has both immediate and thirty-minute delay free recall trials, and a recognition trial. Words were carefully selected so that they would be familiar to all Batswana, even subjects with minimal or no education, and translated easily between Setswana and English. Scores analyzed included the total number of words learned for the 5 trials (TL), immediate free recall (IR), delayed free recall (DR), and recognition (R).

### Trail Making Test A [32]

The Trail Making Test A (TA) assesses psychomotor speed, attention, sequencing, and visual scanning efficiency. This task required participants to connect numbered circles, randomly scattered over a sheet of paper, sequentially from 1 to 25. The score is the time taken to correctly connect the numbered circles.

### Color Trails 2 (CT2) [33]

In addition to the skills required for Trail Making Test A, this test also assesses set-shifting ability. To minimize cultural bias, no letters or written instructions are used. It consists of numbered colored circles from 1 to 25, in bright yellow and pink. Each number is represented twice, once in each color. Using a pencil the subject is required to connect the numbers sequentially, while alternating between the two colors. The score is the time taken to correctly connect the numbered circles.

### Grooved Peg Board Test (GPT)

This test measures fine motor speed and manual dexterity for each hand. Subjects were required to insert small grooved pegs into matched holes on a board. Each hand is tested separately and the score is the time taken to correctly place all of the pegs [34].

### Primary Care Evaluation of Mental Disorders (Prime-MD) [35]

#### Three modules were administered from the Prime-MD

*Mood Module (MM):* The MM is a focused interview, that follows DSM-IV criteria [36], for screening medical patients for current depression, composed of simple “yes” and “no” questions. If five or more of the nine symptoms are present and one of these symptoms is sadness/hopeless or anhedonia, then a diagnosis of MDD is supported. The MM has been successfully used to diagnose depression in patients with HIV and was found to have a sensitivity of 77% and specificity of 84% [37]. In addition, it was previously validated in HIV+ Batswana individuals [38].

*Generalized Anxiety Module (GAM):* The Generalized Anxiety Module consists of 7 simple “yes” and “no” questions aimed at diagnosing Generalized Anxiety Disorder. Subjects must acknowledge feeling anxious and have three or more symptoms of anxiety to meet DSM-IV criteria for a Generalized Anxiety Disorder.

*Alcohol Module (AM):* This inventory is composed of six “yes” and “no” questions about alcohol use during the past six months, aimed at gauging how much alcohol has interfered with activities such as relationships, school/work, and driving. The higher the score, the more alcohol has interfered with daily responsibilities.

### Activities of Daily Living Scale (ADL)

This questionnaire was selected for its wide use and demonstrated validity in studies of medically ill and dementia populations, including HIV subjects in developing countries [39]. It is a 14-item scale measuring physical self-maintenance (e.g., dressing, bathing) and instrumental activities of daily living (e.g., preparing meals, taking medications). Each item is rated on a four-point scale: (1) no difficulty at all; (2) has some difficulty; (3) needs some assistance; (4) can't do at all. Higher scores indicate more impairment in daily functioning.

#### Data Analysis

We performed descriptive analyses and comparisons to examine the demographic and clinical characteristics of patients. All analyses were conducted using StataMP 10.0 (College Station, TX). Correlation coefficients were used to assess relationships between continuous variables. Student's t-test and analysis of variance (ANOVA) were used for comparisons of normally distributed variables. Kruskal-Wallis rank was used for non-normal variables, and Fisher's exact tests and Chi^2^ tests were used for categorical variables. Variables were transformed to normality, as possible. The relative differences in cognitive impairment between the HIV+ and HIV- groups, adjusted for demographic (age, gender, education, and opportunistic infection) and HIV characteristics (nadir/current CD4 count, time since beginning HAART, a detectable viral load), was assessed using regression models. As this was an exploratory study, alpha was set at 0.05 to determine statistical significance. Additionally, the study was designed to detect differences between HIV+ and HIV- subjects in continuous cognitive impairment measures of at least 0.5 standard deviation units.

## Results

The two groups were comparable in age, education, and gender distribution (Table 1). HIV+ patients were more likely to be married whereas HIV- controls were more likely to have chosen to do the study test in Setswana. All of the HIV+ patients were prescribed HAART, having met criteria for HAART initiation as defined in the Botswana National HIV Guidelines [40], and none of the HIV- participants were prescribed HAART.

**Table 1:** Demographic Characteristics of Subjects.

DEMOGRAPHIC CHARACTERISTICS	HIV- CONTROLSUBJECTS(N = 80)	HIV+SUBJECTS(N = 60)	P-VALUE
Age in years (SD)	35.8 (6.7)	37.5 (6.2)	0.11
Total education in years (SD)No educationPrimarySecondaryPost-Secondary	9.3 (4.2)10%18.8%58.8%12.5%	8.6 (4.2)13.3%20%51.7%15%	0.24
Gender (% male)	50%	48.3%	0.87
Marital status:SingleMarried	79.7%20.3%	58.3%41.7%	0.01
Employment status:EmployedUnemployedMonths worked in last year	77.5%22.5%8.7 (4.9)	71.7%28.3%7.6 (5.2)	0.440.13
Language of test administration:SetswanaEnglishBoth	78.8%17.5%3.8%	61.7%36.7%1.7%	0.03

### Laboratory Characteristics

These are summarized in Table 2. Nadir CD4 counts were unavailable for eight HIV+ subjects, so analyses between laboratory values and neuropsychological measures excluded them. Nadir CD4 count was not associated with cognitive impairment (p = 0.3), nor was current CD4 count (p = 0.3). Furthermore, nadir CD4 count was not associated with depression (p = 0.5), anxiety (p = 0.3), or difficulty with ADL (p = 0.14). Similarly, current CD4 count was not associated with any of these measures (depression p>0.99, anxiety p = 0.2, ADL p = 0.12).

**Table 2:** Clinical and Laboratory Characteristics of 60 HIV Infected Subjects (Mean scores and standard deviations in parentheses).

LABORATORY CHARACTERISTICS	PERCENT	MEAN	STANDARD DEVIATION	RANGE
Current CD4 count		416.4	198.5	82.0–955.0
Subjects with current CD4<200	15%			
Nadir CD4		97.6	49.9	4.0–186.0
Time since HIV diagnosis (years)		4.6	2.5	0.5–15.1
Viral Load:<400>400	93.3%6.7%			
On HAART (>3 drugs)	100%			
HAART treatment duration (years)		4.1	2.1	0.1–10.8
Self-reported adherence as “always”	78.3%			
Peripheral neuropathy	40%			
Prior opportunistic infections:TBSyphilis	35%22%			

Ninety-three percent of subjects in our sample had an undetectable viral load (i.e. viral load was <400 copies/mL). Thus, it was not feasible to analyze the data as a continuous outcome. Viral load was therefore examined as a dichotomous outcome (detectable versus undetectable). We found no association between viral load and any of depression, anxiety, or ability to perform ADL, even at a bivariate level.

### Neuropsychological Performance for HIV+ and HIV- Subjects

Table 3 summarizes raw scores (means and standard deviations) on the neuropsychological tests, and also indicates effect sizes comparing the HIV+ group to the uninfected group, adjusted for the effects of age, education, gender, and history of opportunistic infection. Mean t-scores for the neuropsychological tests all showed significant group effects. The HIV+ group was significantly impaired compared to the HIV- group on measures of processing speed (DSC, p<0.01), verbal learning and free recall memory (BAVLT: TL p = 0.01, IR p<0.01, DR p<0.01), but not recognition (R p = 0.64). HIV+ subjects made more errors on the BAVLT, which included false positive (p<0.01) and intrusion errors (p = 0.01).

**Table 3:** Performance on Neuropsychological Measures.

	CONTROLS(N = 80)	HIV+(N = 60)	P-value
NEUROPSYCHOLOGICAL MEASURESProcessing Speed:Symbol Digit Coding (SDC)	52.4 (16.1)	40.8 (17.5)	<0.01
Verbal Learning/Memory (BAVLT):Total Words (T)Immediate Recall (IR)Delayed Recall (DR)Recognition (R)Intrusion Errors (Total)False Positive Recognition Errors	54.3 (12.02)12.1 (2.2)12.4 (2.3)15.3 (0.9)2.7 (4.2)1.5 (2.8)	48.4 (14.84)9.8 (2.3)10.2 (2.8)14.8 (2.2)4.4 (5.3)2.8 (3.5)	0.01<0.01<0.010.640.01<0.01
Fine Motor Speed/Dexterity (GPB):Dominant Hand Time (seconds)Non-dominant Hand Time (seconds)	80.2 (21.7)88.0 (16.8)	86.4 (25.1)102.5 (38.1)	0.040.01
Executive Functions:Color Trails 2 (CT2, Time in seconds)Color Trails 2 (Errors)	123.7 (60.10)0.4 (0.9)	164.9 (98.4)0.9 (1.5)	<0.010.03
Language:Action Fluency (AF, Total Words)	11.6 (3.8)	9.3 (3.9)	<0.01
Psychomotor Speed:Trail Making Test A (TA, seconds)	52.6 (23.9)	68.7 (38.5)	0.01
PSYCHOLOGICAL MEASURES			
PRIME-MDMajor Depressive DisorderSuicidal IdeationGeneralized Anxiety Disorder	28.8%7.5%25%	35%15%41.7%	0.470.180.04

Raw scores, means, standard deviations (in parentheses), and effect sizes on individual neuropsychological test measures for HIV+ and HIV- subjects.

The HIV+ group was also impaired when assessing fine motor speed/dexterity in both dominant (D/GPB, p = 0.04) and non-dominant hands (ND/GPB, p = 0.01), psychomotor speed (TA, p = 0.01), executive functioning (CT2, p<0.01), and language (AF, p<0.01).

### Association Between Neuropsychological Impairment, Alcohol Use and Psychological Measures (Anxiety/Depression)

There was no difference between the two groups with respect to alcohol use (p = 0.6). Subjects in both groups reported very minimal use of alcohol and virtually no participants reported current substance abuse.

Forty-two percent of HIV+ subjects met criteria for Generalized Anxiety Disorder (GAD), which was higher than the 25% in controls (p = 0.04). In contrast, incidence of Major Depressive Disorder (MDD) was similar for the two groups (HIV+ 35%, HIV− 28.8%, p = 0.5), as was level of suicidal ideation (HIV+ 15%, HIV− 7.5%, p = 0.18). There was no difference in neuropsychological performance for depressed (n = 96) and non-depressed subjects (n = 44). These findings will be described in detail elsewhere.

### Definition of Cognitive Impairment and Association with other Factors

According to recently updated research definitions of HAND, Asymptomatic Neurocognitive Impairment (ANI) is defined by performance at least 1 SD below the mean of demographically adjusted normative scores in at least two cognitive areas, with at least 5 cognitive domains measured. Criteria for ANI also require that the impairment: does not occur solely as part of delirium, cannot be explained by other co-morbidities, pre-existing conditions, depression, or substance abuse, and does not interfere with everyday functioning [4]. To be consistent with this definition while account for some non-normality in results achieved by control subjects on grooved peg board testing, the lowest 10^th^ percentile of results achieved by HIV- control subjects was defined as the lower limit of normal performance for the six cognitive-motor measures studied here. Lower performance than this on at least 3 tests (and thus at least two cognitive areas) was defined as impaired to at least the level of ANI. A substantial number of HIV+ subjects tested as cognitively impaired using this definition. Thirty seven percent of HIV+ subjects scored impaired on three or more measures and were defined as cognitively impaired (Table 4). Univariate analysis revealed that cognitive impairment was associated with increasing age, fewer years of education and having been married. Cognitive impairment was not associated with number of years since HIV diagnosis, CD4 count (current or nadir), neuropathy, depression, medication adherence, or alcohol use (Table 5). Multivariate analysis demonstrated, among HIV+ patients, fewer years of education (p = 0.007) and older age (p = 0.002) were independently associated with cognitive impairment (See Table 6).

**Table 4:** HIV+ Subjects Impaired on Neuropsychological Measures.

NEUROPSYCHOLOGICAL MEASURE	CUT OFF	IMPAIRED*
Processing Speed: Digit Symbol Coding	32	32%
Memory: Delayed Recall (BAVLT)	9	37%
Language/Verbal Fluency:Action Fluency	7	38%
Psychomotor Speed: Trails A	78.5	30%
Executive Function: Color Trails 2	221.5	20%
Fine Motor Speed: GPB Dominant Hand	100	22%
Fine Motor Speed:GPB Non-dominant Hand	108.5	25%
Cognitive Impairment	>3 impaired scores	37%

The lower 10^th^ percentile of results achieved by HIV- control subjects was defined as the lower limit of normal performance for 6 cognitive tests and 2 measures of fine motor speed. *Lower performance than this was defined as impaired. HIV+ subjects who scored impaired on three or more tests were defined as cognitively impaired. The cut off results used to differentiate normal and impaired performance are shown here.

**Table 5:** Characteristics of Impaired and Non-Impaired HIV+ Subjects.

HIV+ PATIENT FACTORS	IMPAIRED(N = 22)	NON-IMPAIRED(N = 38)	P VALUE
Age	42 (6.1)	34.8 (4.5)	<0.0001
Education (years)	5.8 (4.1)	10.3 (3.4)	<0.0001
Employment (Full-Time)	16 (73%)	19 (50%)	0.1
Never Married	15 (68%)	34 (89%)	0.04
Neuropathy	8 (36%)	16 (42%)	0.7
Alcohol Use	4 (18%)	8 (21%)	0.8
Years Since HIV Diagnosis	4.6 (1.6)	4.6 (2.9)	0.9
Nadir CD4 (range in parentheses)	109 (4–415)	137 (4–453)	0.1
Current CD4	373 (175–713)	442 (82–955)	0.3
Detectable Virus	2 (9%)	2 (5%)	0.6
Years on HAART	4.4 (1.7)	3.9 (2.3)	0.3
Depression	7 (32%)	14 (37%)	0.7
Non-Adherence	3 (14%)	10 (27%)	0.3
CPE Score	1.95 (0.49)	2.01 (0.62)	0.6

Univariate analysis of associations between patient factors and cognitive impairment for HIV+ subjects, defined as impaired and non-impaired. Mean scores/years and standard deviations (in parentheses).

**Table 6:** Logistic regression model of factors independently associated with cognitive impairment in this cohort.

Variable	Odds ratio	95% Confidence interval	p value
Age in years	1.28	1.1, 1.49	0.002
Years of education	0.76	0.62, 0.93	0.007

(Model R^2^ = 0.40, p<0.0001).

### HAART Regimen and Cognitive Function Among HIV+ Subjects

All HIV+ subjects were on HAART and drug regimens were recorded for 95% of subjects. Seventy-eight percent of patients were on standard first line regimens consisting of two nucleoside (or nucleotide) analog reverse transcriptase inhibitors (one of Zidovudine, Tenofovir or, Stavudine, together with either Lamivudine or Emtricitabine), and either a non-nucleoside analog reverse transcriptase inhibitor (Nevirapine or Efavirenz), or a protease inhibitor (ritonavir-boosted Lopinavir). In addition, two patients were using Abacavir as a fourth drug. Seventeen percent of patients were using second line therapy, which in all cases consisted of zidovudine, lamivudine and lopinavir/ritonavir. Each drug was given a CNS penetration score [41] to determine whether the total CNS penetration score of the regimens was associated with cognitive impairment. Antiviral drugs with high ability to penetrate the blood-brain barrier were given a penetration score of 1 (nevirapine, lopinavir/ritonavir, abacavir, zidovudine), drugs with intermediate ability were given a score of 0.5 (stavudine, lamivudine, emtricitabine, efavirenz), and poor ability was scored 0 (tenofovir). The mean penetration score of HAART regimens used by patients was 2 (range 0.5–3.5). There was no difference (p = 0.6) in CNS penetration scores for patients who were cognitively impaired and patients who were not cognitively impaired (Table 5)

### Activities of Daily Living, Adherence, and Employment

Slightly over 21% of HIV+ subjects reported difficulty adhering to ARV regimens, which they attributed exclusively to memory difficulties. Subjects who reported problems with adherence did not differ on any of the demographic variables of age (p = 0.2), education (p = 0.5), gender (p = 0.2), or marital status (p>0.99). Despite subjective complaints of difficulty remembering to take medication, these subjects were not more impaired on objective memory measures on the BAVLT than patients reporting high levels of medication adherence (TL p = 0.8, IR p = 0.4 DR p = 0.7). Details of the ADL results are described in detail elsewhere.

## Discussion

There are two main findings in this study. *First*, HIV+ subjects were impaired on neuropsychological measures when compared to demographically matched controls for all cognitive-motor ability areas, which included processing speed, verbal learning/memory, language, psychomotor speed, executive function, and fine motor speed (dominant and non-dominant hands). Using a definition of impairment as scoring below the 10^th^ percentile of results achieved by control subjects on three or more measures of cognition and/or fine motor speed, a significant proportion (37%) of HIV+ subjects were cognitively impaired. *Second*, there was no difference in CNS penetration scores for impaired and non-impaired HIV+ subjects, but those who were cognitively impaired were older and had less education than subjects who were not cognitively impaired.

This study has several limitations, which include a relatively small sample size, the use of a symptom scale to identify peripheral neuropathy rather than a neuromedical assessment, and an ADL scale that may not have been sensitive to detect subtle changes in activity levels in this Batswana cohort.

The prevalence of cognitive impairment (37%) is similar to prior research, which used the International HIV Dementia Scale (IHDS) and found 38% of HIV+ Batswana subjects, 97.5% of whom were on HAART, to be cognitively impaired [27]. We had anticipated that cognitive impairment would be associated with regimens with lower CNS penetration scores since prior studies have demonstrated that regimens with higher CNS penetration scores are associated with better control of CNS viral load [41] and better cognitive outcomes [42]. But there was no difference in CNS penetration scores between HIV+ impaired and unimpaired subjects.

All of the HIV+ subjects were prescribed HAART, which was expected to suppress HIV viral loads and improve current CD4 counts. Most patients had undetectable plasma HIV viral loads. Thus we hypothesized that neuropsychological status would be related to nadir CD4, but not current CD4. The results did not confirm this, and neither lower nadir nor current CD4 T-cell counts were a risk factor for cognitive impairment. It is possible that CD4 count (nadir and current) was not associated with cognitive impairment because most subjects in this study had achieved normal or near-normal CD4 counts, and subjects with a known history of CNS opportunistic infections were excluded.

In the current study, older age and fewer years of education within HIV+ subjects were associated with cognitive impairment. A recent study of HAND in South Africa also found a significant association with lower levels of education and older age [18]. Future large scale studies of healthy Batswana with diverse ages and educational backgrounds will be needed to develop norms that can be used to confidently classify HIV-related impairments in individual cases.

Verbal learning and free recall memory were impaired for HIV+ subjects, but recognition memory was not. This is consistent with prior studies showing impaired free recall due to a retrieval deficit, but intact recognition memory [43]. Qualitatively, HIV+ subjects demonstrated executive dysfunction on the BAVLT, as illustrated by more intrusion and false positive errors, which is reflective of the frontal-striatal brain damage that is typical of HIV. This pattern of impairment is in agreement with previous studies in sub-Saharan Africa [13], [14], [44].

From prior work in the same setting, we anticipated a relatively high prevalence of depression in HIV+ individuals [38]. However, we were surprised by the similarly high level of depression and suicidal ideation in the demographically matched, uninfected control group. The double rate of suicidality in HIV+ subjects is clinical meaningful, but the study lacked the power to find it statistically significant. These data suggest that depression is common in both HIV+ and HIV- adults in this setting and that HIV infection itself may not be the explanation (or the only explanation) for the high rate of depression previously described in HIV+ adults in Botswana. It has been suggested that rapid changes in Batswana society have contributed to the high rate of depression and suicide [45]. This includes changes in gender roles as women have gained access to education, a decrease in marriage, and employment patterns that have led to a steady migration away from the traditional patriarchal family structure to a more isolated life-style in urban settings.

HIV+ subjects did not report difficulty with routine daily activities on the ADL scale, and were as likely to be employed as the HIV- control subjects. One possible explanation for this is that the neuropsychological deficits do not have the same relevance to everyday activities for HIV+ individuals in Botswana, as compared to US studies where even mild cognitive impairment has been shown to adversely impact vocational activities [6]. An alternative explanation is that the ADL scale used in this study may not be valid in developing countries, such as Botswana, where people have very different educational, cultural, and linguistic backgrounds from those in the United States, Europe, and Australia. Other researchers have pointed out that until more culturally appropriate ADL measures have been validated for a particular population, it may be more reliable to focus on objective neuropsychological test performance, especially for comparison across international settings [23]. HIV-related changes in cognitive ability may be much less likely to cause obvious functional decline in cultures that have fewer demands on such cognitive abilities [4]. Thus the addition of an asymptomatic category of neurocognitive impairment, such as ANI, may be particularly important in these types of settings to prevent such cases of cognitive impairment from being missed. The recognition of mild cognitive impairment in asymptomatic individuals may encourage more frequent neurologic follow-up for individuals of ANI, help with decisions related to the initiation of ARVs independent of biomarkers, and guide the selection of ARV regimens.

In conclusion, neuropsychological impairment in this Batswana sample was associated with HIV infection, and the pattern was similar to neuropsychological studies previously carried out in developed and developing countries, including sub-Saharan Africa. Within the HIV+ group, those who met criteria for cognitive impairment were older and had less education than patients who were not cognitively impaired. These findings indicate that HAND is likely to be important among HAART-treated HIV individuals in developing countries.
